# 1670. Activity of Ceftolozane/Tazobactam and Comparators Against Clinical MDR and DTR *Pseudomonas aeruginosa* isolates – SMART United States 2018-2020

**DOI:** 10.1093/ofid/ofac492.1300

**Published:** 2022-12-15

**Authors:** Sibylle Lob, Meredith Hackel, Fakhar Siddiqui, Karri A Bauer, Charles A DeRyke, Katherine Young, Mary Motyl, Daniel F Sahm

**Affiliations:** Merck & Co., Inc., Schaumburg, Illinois; IHMA, Schaumburg, Illinois; Merck & Co., Inc., Schaumburg, Illinois; Merck Research Laboratories, Kenilworth, New Jersey; Merck Research Laboratories, Kenilworth, New Jersey; Merck, Rahway, New Jersey; Merck, Rahway, New Jersey; IHMA, Schaumburg, Illinois

## Abstract

**Background:**

Antimicrobial resistance of *P. aeruginosa* to commonly used β-lactams is typically higher in isolates collected from ICU patients and those with hospital-acquired infections. *P. aeruginosa* with multidrug-resistance (MDR) or difficult-to-treat resistance (DTR) is especially challenging as clinicians have limited treatment options. Ceftolozane/tazobactam was specifically developed to provide enhanced antibacterial activity against *P. aeruginosa*. We evaluated the activity of ceftolozane/tazobactam (C/T) and comparators against clinical *P. aeruginosa* isolates by ward type and length of hospital stay at time of specimen collection, including against MDR and DTR isolates.

**Methods:**

In 2018-2020, 24 clinical labs participated in the global SMART surveillance program in the United States (US) and each collected up to 250 consecutive gram-negative pathogens per year from patients with bloodstream, intraabdominal, lower respiratory tract, and urinary tract infections. MICs were determined using CLSI broth microdilution and interpreted with CLSI breakpoints.

**Results:**

C/T and amikacin were the only studied agents with activity >95% against all isolates in all strata, with only small differences between the strata (Table). Susceptibility to commonly used β-lactams was 4-8 percentage points lower among isolates collected ≥48 hours post-admission than < 48h and from patients in ICUs compared to non-ICU wards. Correspondingly, the prevalence of MDR and DTR isolates was higher among isolates collected ≥48 hours post-admission (14.6% and 7.3%, respectively) than < 48h (10.5%/6.1%) and those collected from ICU patients (16.1%/7.2%) than non-ICU (10.0%/5.8%). The pattern of lower susceptibility among isolates collected ≥48h post-admission or in ICUs was less clear when the MDR and DTR subsets were analyzed. C/T remained active against ≥72% of MDR and ≥68% of DTR isolates, 7-31 percentage points higher than ceftazidime/avibactam.

**Figure:**
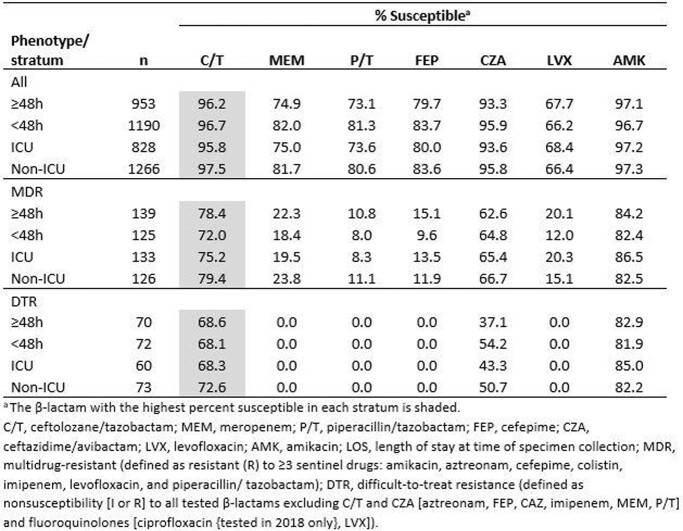

**Conclusion:**

Based on these *in vitro* data, C/T represents a treatment option for patients with infections caused by MDR and DTR *P. aeruginosa*, regardless of time to infection or treatment in the ICU.

**Disclosures:**

**Fakhar Siddiqui, MD, MBA**, Merck & Co., Inc.: employee|Merck & Co., Inc.: Stocks/Bonds **Karri A Bauer, PharmD**, Merck & Co., Inc. Merck Research Laboratories: Stocks/Bonds **Charles A. DeRyke, PharmD**, Merck & Co., Inc. Merck Research Laboratories: Stocks/Bonds **Katherine Young, M.S.**, Merck & Co., Inc.: Stocks/Bonds.

